# Mesial temporal shape asymmetry as a neuro-imaging correlate of epilepsy in mild cognitive impairment and dementia

**DOI:** 10.1093/braincomms/fcag179

**Published:** 2026-05-18

**Authors:** Ifrah Zawar, Shen Zhu, Mark Quigg, Jaideep Kapur, Alice D Lam, Rani A Sarkis, Anelyssa D’Abreu, Carol Manning, P Thomas Fletcher

**Affiliations:** Department of Neurology, Comprehensive Epilepsy Program, University of Virginia, Charlottesville, VA 22908, USA; Department of Computer Science, University of Virginia, Charlottesville, VA 22908, USA; Department of Neurology, Comprehensive Epilepsy Program, University of Virginia, Charlottesville, VA 22908, USA; Department of Neurology, Sleep Center, University of Virginia, Charlottesville, VA 22908, USA; Department of Neurology, Comprehensive Epilepsy Program, University of Virginia, Charlottesville, VA 22908, USA; Department of Neurology, Massachusetts General Hospital, Harvard Medical School, Boston, MA 02114, USA; Department of Neurology, Brigham and Women’s Hospital, Harvard Medical School, Boston, MA 02115, USA; Department of Neurology, Memory Disorders Program, University of Virginia, Charlottesville, VA 22908, USA; Department of Neurology, Memory Disorders Program, University of Virginia, Charlottesville, VA 22908, USA; Department of Computer Science, University of Virginia, Charlottesville, VA 22908, USA; Department of Electrical Engineering, University of Virginia, Charlottesville, VA 22908, USA

**Keywords:** seizures, cognition, dementia, hippocampus, amygdala

## Abstract

Epilepsy is a common comorbidity of Alzheimer’s disease (AD) and related dementias (ADRD). Both epilepsy and ADRD affect the mesial temporal structures in volume and morphology. Nonetheless, mesial temporal shape and volume asymmetry remain understudied in persons with dementia and mild cognitive impairment, collectively called persons with cognitive impairment (PWCI) and co-morbid epilepsy. We investigated hippocampal and amygdalar left-to-right shape and volume asymmetry in AD (AD_Epi) and non-AD (nonAD_Epi) with co-morbid epilepsy. This multicentre study included participants from 39 US Alzheimer’s disease centres from 9/2005 to 12/2021. We categorized participants into group 1: PWCI with epilepsy (subclassified into AD_Epi and nonAD_Epi); group 2: PWCI without epilepsy (subclassified into AD_NoEpi and nonAD_NoEpi); and group 3: healthy controls (HC). We used a fixed-ratio, optimal propensity score matching to match group 1 participants to groups 2 and 3. Matching was based on age, sex and type of dementia (AD versus nonAD) for group 2 and age and sex for group 3. We used FreeSurfer for MRI segmentation of hippocampi and amygdalae. For volume asymmetry, we subtracted the right volume from the left and divided it by the total volume of the structure (right + left). For shape asymmetry, we calculated a 512-point shape model using left and flipped right hippocampi and, similarly, a 256-point shape model for amygdalae. Next, we calculated the point-by-point shape asymmetry between left and right hippocampi and amygdalae in a normal direction for each participant. Multivariable linear models were used to compare volume and shape asymmetry among groups after adjusting for age, sex, total intracranial volume and dementia severity. We compared AD_Epi versus AD_NoEpi, nonAD_Epi versus nonAD_NoEpi, AD_Epi versus HC, nonAD_Epi versus HC, AD_NoEpi versus HC and nonAD_NoEpi versus HC. Analyses were adjusted for multiple comparisons. A total of 703 participants were included [391(55.62%) female, average age: 70.78 years]. These included 35 AD_Epi, 28 nonAD_Epi, 183 AD_NoEpi, 137 nonAD_NoEpi and 320 HC. For shape analyses, AD_Epi showed greater left-to-right asymmetry (Left smaller than right) in hippocampal tail compared with HC. Those with nonAD_Epi demonstrated greater left–to-right asymmetry in the hippocampal head compared with both nonAD_NoEpi and HC. AD_NoEpi had an asymmetrically smaller left hippocampal head and right amygdala than HC. We found no group differences in volume asymmetries. Our study found left smaller than right hippocampal head asymmetry in non-Alzheimer’s dementia with epilepsy and left smaller than right hippocampal tail asymmetry in Alzheimer’s disease with epilepsy. These findings suggest that hippocampal shape asymmetry may serve as a neuroimaging correlate of epilepsy in ADRD.

## Teaser

Zawar *et al*. report that individuals with Alzheimer’s disease and related dementias who also have epilepsy show distinct hippocampal shape asymmetries. Specifically, the left hippocampal head was asymmetrically smaller than the right in non-Alzheimer’s dementias with epilepsy, while the left hippocampal tail was asymmetrically smaller in Alzheimer’s disease with epilepsy, suggesting epilepsy-related regional hippocampal vulnerability.

## Graphical Abstract

**Graphical Abstract:**
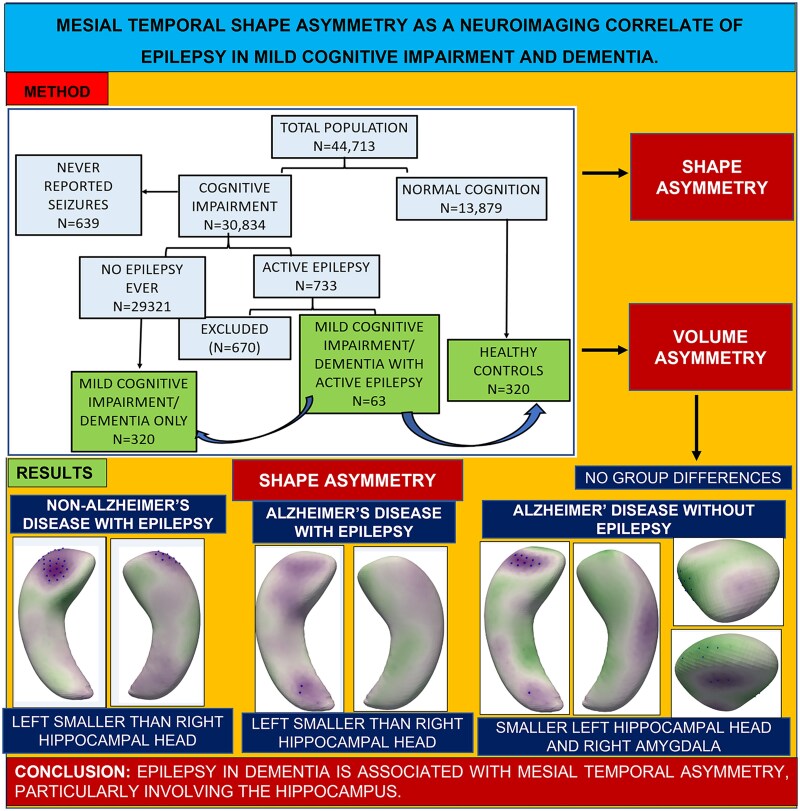

## Introduction

Epilepsy is a common co-morbidity of Alzheimer’s disease (AD) and related dementias (ADRD).^1-7^ Clinical seizures are observed in 10–64% of people with mild cognitive impairment (MCI) and dementia collectively called persons with cognitive impairment (PWCI).^1-4^ People with ADRD face a 6 to 8-fold greater risk of epilepsy compared with the general population.^1-4^ People with ADRD and co-morbid epilepsy experience a more severe disease progression, more significant neuronal damage, higher mortality and worse functional and cognitive morbidity.^8-17^ Despite the high incidence, mortality and morbidity of epilepsy, there have been few studies investigating the neuro-imaging correlates of epilepsy risk in neurodegenerative disorders. There is limited understanding of the distinct neuro-imaging biomarkers of co-morbid epilepsy in AD and non-AD neurodegenerative disorders.

The mesial temporal lobe structures, the hippocampus and the amygdala, are frequently affected early in both PWCI and epilepsy.^18,19^ People with epilepsy often demonstrate hippocampal and amygdalar atrophy.^20-22^ Similarly, people with ADRD have mesial temporal lobe atrophy.^18,19^ The amygdala and hippocampus are also regions of early deposition of pathologic protein in AD.^23,24^

Although healthy mesial structures may demonstrate slight asymmetry, ADRD and epilepsy may affect mesial temporal symmetry to a significantly greater degree.^25,26^ Emerging evidence suggests some mesial temporal asymmetry may be present in AD.^25,26^ Recently, we demonstrated hippocampal shape asymmetry as a reliable distinguishing feature between AD without epilepsy compared with healthy controls.^27^ Epilepsy in ADRD also tends to be focal and, therefore, unilateral and lateralized.^28^ Comorbid epilepsy is also associated with asymmetric neuropathological changes, including tau and amyloid in AD.^28^ These findings suggest that mesial temporal asymmetry may serve as a useful biomarker of comorbid epilepsy in ADRD, and there may be a structural correlation that underlies the asymmetry of epilepsy in ADRD or vice versa.^26^

Nonetheless, hippocampal and amygdalar shape and volume asymmetry remain understudied in ADRD and co-morbid epilepsy, and the impact of the intersection between the two diseases on the hippocampus and amygdalar shape remains unexplored. We investigated the asymmetry of the hippocampus and amygdala shape and volume to identify distinct morphological patterns of asymmetry in people with AD (AD_Epi) and nonAD dementias (non-AD_Epi) with co-morbid epilepsy compared with those without comorbid epilepsy. Understanding neuro-imaging biomarkers of epilepsy in ADRD may help early identification and timely treatment of epilepsy.

## Materials and methods

### Study design

This study utilized anonymized cross-sectional multicentre data from the National Alzheimer’s Coordinating Centre, comprising participants recruited and followed over time at 39 Alzheimer’s Disease Research Centres (ADRC) throughout the United States.^29-31^ The data in our study span from September 2005 to December 2021. This research adhered to the Strengthening the Reporting of Observational Studies in Epidemiology (STROBE) reporting guidelines.

Written informed consent and ethical board approvals are secured and maintained at the ADRC centres. Our institution deemed the study exempt from ethics board review because it utilized anonymized data.

### Participants

#### Epilepsy diagnosis

In this study, epilepsy is defined as patients having recurrent seizures at least in the previous 12 months and those requiring active treatment with anti-seizure medications. This definition meets the clinical epilepsy definition of the International League Against Epilepsy (ILAE).^32^ This diagnosis was determined based on participant and caregiver interviews, clinical observation and electronic medical records. Seizure status was evaluated at the initial visit and then re-evaluated at each subsequent visit.

#### Participant selection with ADRD and co-morbid epilepsy (group 1)

**Figure 1:**
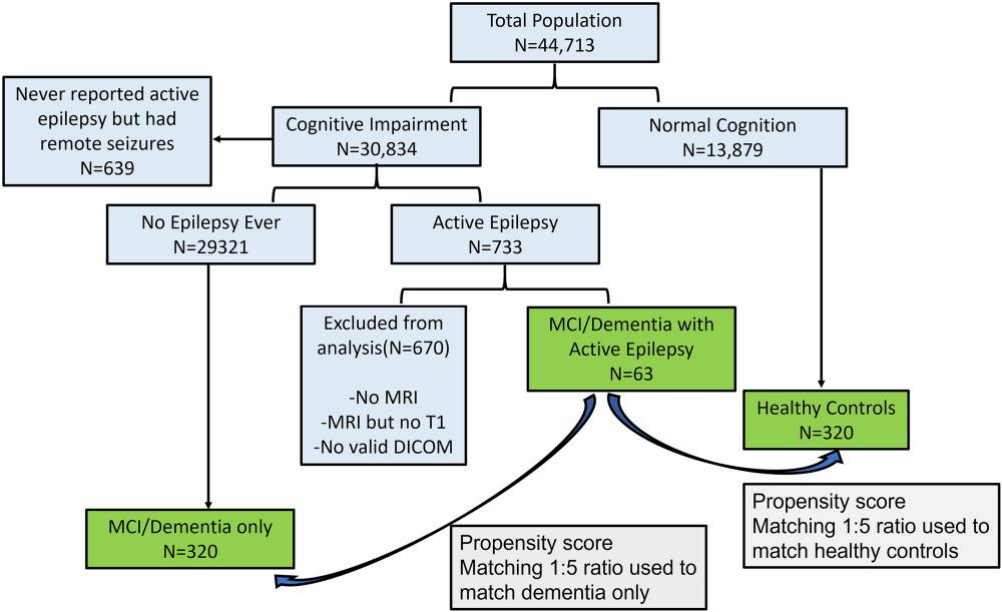
**Study flow chart.** The flow chart demonstrates that those with cognitive impairment were separated into those with active epilepsy. Among active epilepsy, those with a valid analysable DICOM (Digital Imaging and Communications in Medicine) MRI (Magnetic Resonance Imaging) T1 scan were included as Group 1. Using propensity score matching of 1:5, these participants were matched with group 2: 320 participants with MCI or dementia, and group 3: 320 healthy controls. Group 2 participants were matched on age, sex and dementia subtype, and group 3 participants were matched on age and sex.

Figure 1 shows the *Study Flow Chart*. First, we selected participants with clinically diagnosed ADRD-related MCI or dementia at enrolment or during the study follow-up. Then, among the cognitively impaired, we selected participants who had at least one visit (enrolment or follow-up) where they reported active seizures. Within those, all participants who had an MRI with a valid high-resolution T1 sequence in analysable DICOM format were included. These participants were further subclassified into those with AD and co-morbid epilepsy (AD_Epi) and those with non-AD and co-morbid epilepsy (nonAD_Epi).

#### Participant selection with ADRD without epilepsy (group 2) and healthy controls (group 3)

Next, we matched ADRD with epilepsy (group 1) with ADRD without epilepsy and healthy controls (HC). Group 2 (ADRD without epilepsy) was further subclassified into those with AD (AD_NoEpi) and those with non-AD (nonAD_NoEpi). Group 3 included healthy controls (HC) with no cognitive impairment and no history of seizures or epilepsy.

### Procedures

#### Participant matching

We utilized a 1:5 fixed-ratio, optimal propensity score matching to select people with ADRD without epilepsy and HC. Propensity score matching was based on age, sex and type of cognitive impairment (AD versus non-AD) for ADRD without epilepsy and age and sex for HC. Propensity score matching is a statistical technique that reduces bias between the cases and controls.^33^ Because the number of participants with epilepsy was significantly smaller compared with other groups, a 1:5 ratio was utilized instead of a 1:1 ratio to increase study power and precision. Optimal matching focuses on creating the best possible pairings between cases and control units based on minimizing the total distance across all matches.^33^ This method was chosen because it offers several key benefits, particularly in observational studies aiming to estimate causal effects. This leads to better homogeneity among the covariates, reduced variance, increased precision and reduced overall bias.^33^

#### Other variables

Other variables included age (continuous, years), *sex* (categorical, female versus male), *race* (categorical, dichotomized to white and non-white), ethnicity (Hispanic versus non-Hispanic), *education* (total years, integer scale continuous) and severity of cognitive impairment (CDR-global and CDR sum of boxes).

#### Mesial temporal segmentation with FreeSurfer

The imaging dataset consisted of raw DICOM files. After converting DICOM files into NIfTI files, we utilized FreeSurfer^34^ to automate the segmentation and parcellation process. We used FreeSurfer version 7.2.0^34^ for cortical parcellation and subcortical segmentation according to the Desikan-Killiany atlas and RB_all_2020-01-02 atlas, respectively. RB_all_2020-01-02 is an updated version of RB_all_2008-03-26.gca, which is FreeSurfer’s probabilistic atlas for subcortical segmentation,^34^ containing learned spatial and intensity priors from manually labelled brains. It guides the aseg algorithm in assigning anatomical labels to each voxel.

We extracted the hippocampus and amygdala labels from the final FreeSurger results from the MRI scans for our 512 or 256-point shape asymmetry models and morphological analysis.

#### Quality control

Visual inspection of shape segmentations and consistency checks across subjects was used to ensure that any variability observed was due to true biological differences rather than artefacts.

Harmonization procedures often aim to reduce unwanted technical variability but can also inadvertently remove biologically meaningful variations. Shape models primarily analyse morphological features that are less sensitive to intensity variation between MRI scanners than voxel-based analyses. Previous research in shape modelling has shown that shape-based metrics exhibit greater robustness across various MRI acquisition parameters and are the most stable neuroimaging feature across MRI scanners,^35^ supporting the approach of not harmonizing the data for this specific analysis. Since our primary outcome was shape models, harmonization was deferred.

### Statistical analysis

#### Data summary

We used R [version (4.2.0), R Foundation for Statistical Computing, Vienna, Austria] and Python for statistical analyses.

#### Baseline analysis

Baseline categorical scaled clinical characteristics were summarized by frequencies (N) and proportion (%), while median and interquartile ranges summarized continuous baseline characteristics. Relationships among the three groups and the baseline characteristics (i.e. age, sex, race, education) and cognition were examined by Pearson’s chi-squared or Fisher’s exact test for categorical variables and the *t*-test, ANOVA, or Wilcoxon Rank Sum test for continuous variables as deemed appropriate. The details of the tests used are noted in the Table footnote.

#### Primary and secondary outcome analysis

The primary outcome was mesial temporal structure (hippocampi and amygdalae) cumulative, point-by-point shape asymmetry in AD_Epi and non-AD_Epi compared with other groups.

In the secondary outcome, we also investigated the mesial temporal structure (hippocampi and amygdalae) volume asymmetry among the groups.

#### Shape asymmetry

To investigate hippocampal and amygdalar shape asymmetry and understand the fine-grained morphological asymmetry differences between ADRD with and without epilepsy, we utilized a point distribution model to represent the shape of the hippocampus and amygdala.^36^

Our method of point-wise quantification and analysis of hippocampal asymmetry^27^ to localize areas with significant asymmetry differences was developed in our earlier study. We used the particle correspondence optimization method of ShapeWorks^36^ to build our model. ShapeWorks is a tool designed to create particle-based shape models from anatomic structures.^27^ This approach simultaneously optimized left and right hippocampi and amygdalar correspondences within and across subjects.^27^

We started from binary segmentation volumes as input, and used ShapeWorks in the ‘groom’ stage to prepare the data. In the grooming pipeline, we applied antialiasing to smooth the foreground/background interface, followed by resampling to isotropic voxel spacing so that all participants were in the same resolution space. After these steps, we converted to a signed-distance transform and applied a mild Gaussian blur for numerical stability.

We calculated a 512-point shape model using both left hippocampi and flipped right hippocampi along the sagittal plane. Next, we calculated the asymmetry between left and right hippocampi along the normal direction (perpendicular to the tangent plane to each point) at each of these 512 points for each participant. By subtracting the right side from the left side, we obtained a directional change from the left to the right side for each point (directional left-to-right asymmetry). We repeated the same analyses for amygdalae using a 256-point shape model suitable for smaller volumes.

Finally, multiple linear regression models were used to compare asymmetry among the groups to account for covariates that may impact hippocampal and amygdalar shape asymmetry. All AD_Epi versus AD_NoEpi and NonAD_Epi versus NonAD_NoEpi shape models were adjusted for covariates of age, sex, total intracranial volumes and dementia severity at the nearest visit measured via CDR-global. AD_Epi versus HC, nonAD_Epi versus HC, AD_NoEpi versus HC and nonAD_NoEpi versus HC comparison linear models for shape were adjusted for covariates of age, sex and total intracranial volumes. Similarly, we investigated inherent asymmetry among HC to confirm that our findings are not just explained by normal expected differences among hemispheres in HC but are truly related to the disease process under investigation. For each HC, we first calculated directional asymmetry vectors by subtracting the left and right shape representations. Then we projected each asymmetry vector onto the normal vector at a given point. Linear models were built, adjusting for covariates of age at the time of scan, sex and total intracranial volume.

**Figure 2:**
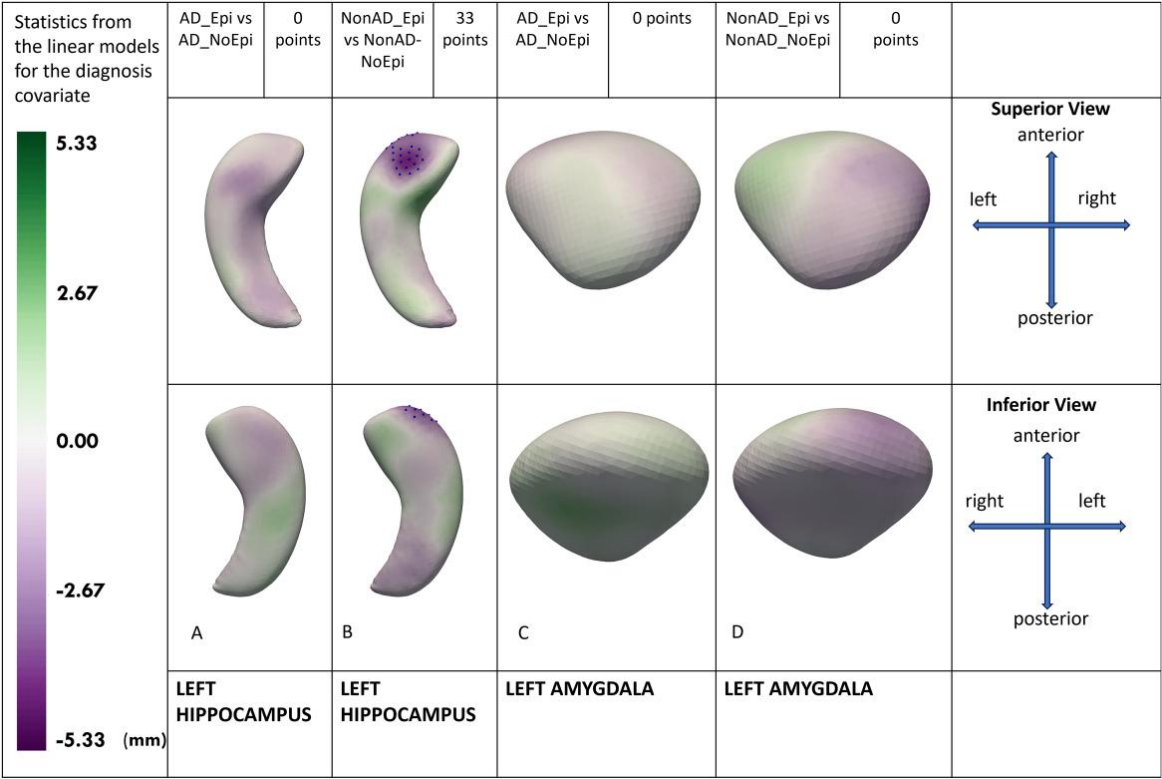
**Mesial temporal shape asymmetry in ADRD (Alzheimer’s disease and related dementias) with and without epilepsy.** For shape asymmetry, we calculated a 512-point shape model using left and right hippocampi, and a 256-point shape model for amygdalae. Next, we calculated the point-by-point shape asymmetry between left and right hippocampi and amygdalae in a normal direction for each participant. Multivariable linear models were used to compare volume and shape asymmetry among groups after adjusting for age, sex, total intracranial volume and dementia severity. The green colour corresponds to positive values and implies that left-sided structures are larger than right-sided structures. Purple colour corresponds to negative values and implies that left-sided structures are smaller than right-sided structures. Statistically significant points are shown as dots on the figures. All visualizations are provided on the left-sided structures. **(2A)**: AD (Alzheimer’s Disease) with epilepsy is compared with AD without epilepsy, identifying no significant differences in the hippocampal shape asymmetry. **(2B)**: Non-AD (Non-Alzheimer’s Disease) with epilepsy is compared with non-AD without epilepsy, identifying 33 significant points demonstrating hippocampal shape asymmetry with smaller left hippocampal head compared with right in the non-AD with epilepsy group. **(2C)**: AD with epilepsy is compared with AD without epilepsy, identifying no significant shape asymmetry in the amygdala. **(2D):** Non-AD with epilepsy is compared with non-AD without epilepsy, identifying no significant shape asymmetry in the amygdala.

**Figure 3:**
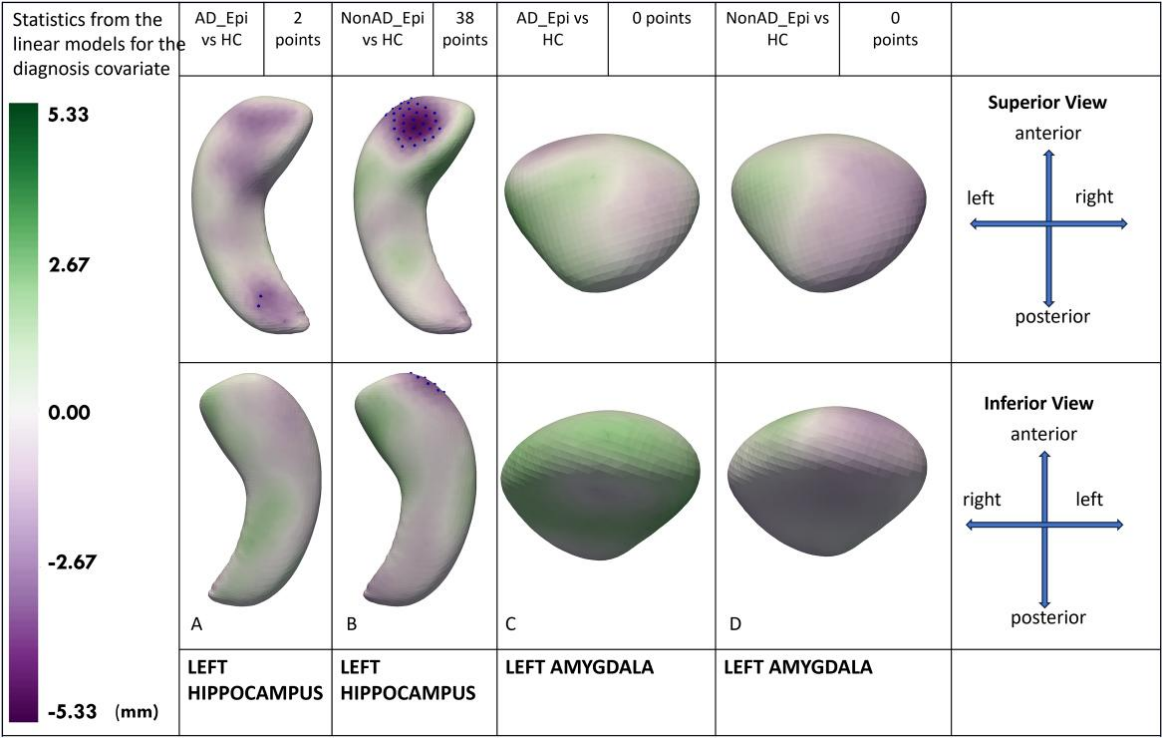
**Mesial temporal shape asymmetry in ADRD (Alzheimer’s disease and related dementias) with epilepsy and healthy controls.** For shape asymmetry, we calculated a 512-point shape model using left and right hippocampi, and a 256-point shape model for amygdalae. Next, we calculated the point-by-point shape asymmetry between left and right hippocampi and amygdalae in a normal direction for each participant. Multivariable linear models were used to compare volume and shape asymmetry among groups after adjusting for age, sex, total intracranial volume and dementia severity. The green colour corresponds to positive values and implies that left-sided structures are larger than right-sided structures. Purple colour corresponds to negative values and implies that left-sided structures are smaller than right-sided structures. Statistically significant points are shown as dots on the figures. All visualizations are provided on the left-sided structures. **(3A)**: AD (Alzheimer’s Disease) with epilepsy is compared with healthy controls, identifying two significant points demonstrating a small left hippocampal tail compared with the right in the AD with epilepsy group. **(3B)**: Non-AD (Non-Alzheimer’s Disease) with epilepsy is compared with healthy controls, identifying 38 significant points demonstrating hippocampal shape asymmetry with a smaller left hippocampal head compared with the right in the non-AD with epilepsy group. **(3C)**: AD with epilepsy is compared with healthy controls, identifying no significant shape asymmetry in the amygdala. **(3D):** Non-AD with epilepsy is compared with healthy controls, identifying no significant shape asymmetry in the amygdala.

**Figure 4:**
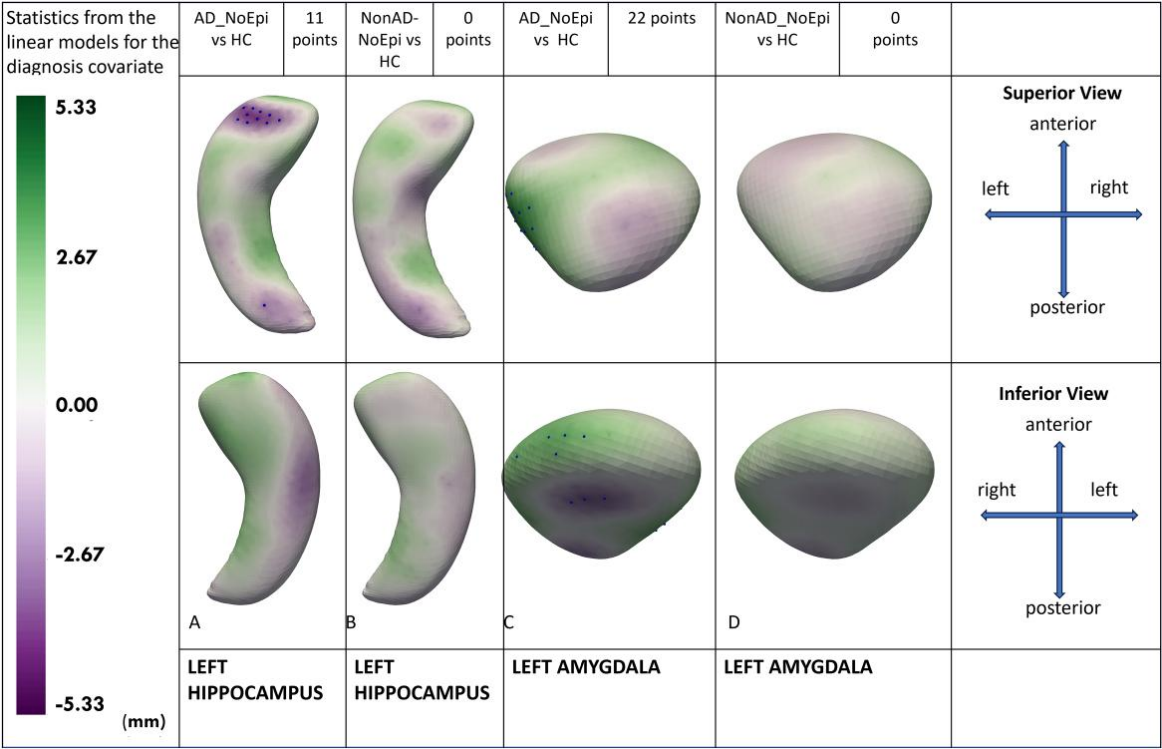
**Mesial temporal shape asymmetry in ADRD (Alzheimer’s disease and related dementias) without epilepsy and healthy controls.** For shape asymmetry, we calculated a 512-point shape model using left and right hippocampi, and a 256-point shape model for amygdalae. Next, we calculated the point-by-point shape asymmetry between left and right hippocampi and amygdalae in a normal direction for each participant. Multivariable linear models were used to compare volume and shape asymmetry among groups after adjusting for age, sex, total intracranial volume and dementia severity. The green colour corresponds to positive values and implies that left-sided structures are larger than right-sided structures. Purple colour corresponds to negative values and implies that left-sided structures are smaller than right-sided structures. Statistically significant points are shown as dots on the figures. All visualizations are provided on the left-sided structures. **(4A)**: AD (Alzheimer’s Disease) without epilepsy is compared with healthy controls, identifying 11 significant points demonstrating a small left hippocampal head and tail compared with the right in the AD without epilepsy group. **(4B)**: Non-AD (Non-Alzheimer’s Disease) without epilepsy is compared with healthy controls, identifying no significant shape asymmetry in the hippocampus. **(4C)**: AD without epilepsy is compared with healthy controls, identifying 22 significant points demonstrating a smaller right lateral amygdala compared with the left in AD without epilepsy. **(4D):** Non-AD without epilepsy is compared with healthy controls, identifying no significant shape asymmetry in the amygdala.

For 512 points (hippocampi) or 256 points (amygdalae), all *P*-values were adjusted using false discovery rate correction for multiple comparisons. False discovery rate correction was used because of the huge number of statistical tests (512 × 6 = 3072 for the hippocampus and 256 × 6 = 1536 for the amygdala). All statistically significant mean hippocampal and amygdalar surface points were utilized to elucidate localized morphological differences between groups. The points in Figs 2–4 show statistically significant differences. The green colour corresponds to positive values and implies that left-sided structures are larger than right-sided structures. Purple colour corresponds to negative values and implies that left-sided structures are smaller than right-sided structures. Statistically significant points are shown as dots on the figures.

#### Volume asymmetry

To calculate hippocampal volume asymmetry, the right hippocampal volume was subtracted from the left hippocampal volume for each group, and the result was divided by the sum of the right and left hippocampal volumes. Similarly, amygdala volume asymmetry was calculated by subtracting the right amygdalar volume from the left amygdalar volume, and the result was divided by the sum of the right and left amygdalar volumes.

Volume asymmetries were initially compared using *t*-tests to determine unadjusted *P*-values. Linear models were then constructed to evaluate hippocampal and amygdala asymmetries while adjusting for covariates. For comparisons between AD_Epi and AD_NoEpi and nonAD_Epi and nonAD_NoEpi, the linear models adjusted for age, sex, total intracranial volume and dementia severity (measured as CDR-global at the nearest visit) to obtain adjusted *P*-values. Similarly, comparisons of AD_Epi versus HC, nonAD_Epi versus HC, AD_NoEpi versus HC and nonAD_NoEpi versus HC were adjusted for age, sex and total intracranial volume to calculate adjusted *P*-values.

#### Comparison groups

We compared the hippocampal and amygdalar shape and volume asymmetry differences among (i) AD_Epi and AD_NoEpi, (ii) NonAD_Epi and nonAD_NoEpi, (iii) AD_Epi with HC, (iv) nonAD_Epi with HC, (v) AD_NoEpi with HC and (vi) nonAD_NoEpi with HC.

### Sensitivity analyses

In sensitivity analyses, all the models above were adjusted for MRI field strength and voxel volume, in addition to age, sex, total intracranial volume, and/or cognition.

#### Missing data

There was complete data available for CDR-Global. For other individual variables, the missing data were minimal (<3%). Consequently, no imputation was necessary for missing predictor variable information. Instead, we excluded missing data from analyses. All analyses used two-sided *P*-values, with a significance threshold set at 0.05.

## Results

### Clinical characteristics

A total of 63 participants were identified who had ADRD with co-morbid epilepsy (Group 1) and a valid, analysable DICOM scan (Fig. 1). These participants were matched with 320 ADRD participants without epilepsy (Group 2) and 320 healthy controls (Group 3) using 1:5 fixed ratio optimal propensity score matching. Thus, 703 participants were included for analysis. The three groups included 35 AD_Epi, 28 nonAD_Epi, 183 AD_NoEpi, 137 nonAD_NoEpi and 320 HC (Detailed characteristics are noted in Supplemental Table 1).

The average age of participants at MRI acquisition was 70.78 years and 55.62% (*N* = 391) were female. The three groups were comparable in age, sex and race (Table 1). The groups differed in ethnicity (Hispanics in Group 1: 14%, Group 2: 12%, Group 3: 5%, *P* = 0.0002) and education (Mean Group 1: 15.1, Group 2: 14.5, Group 3: 16.1, *P* < 0.0001). Cognitive impairment subtypes (AD versus nonAD) and cognition were comparable in Group 1 and Group 2 (Table 2). Among nonAD_Epi and nonAD_NoEpi, subtypes of neurodegenerative disorders were also comparable (Supplemental Table 2).

**Table 1:** Baseline demographics

Characteristics among the 3 groups		Group 1 (*N* = 63) Seizures + MCI/dementia	Group 2(*N* = 320) MCI/Dementia only	Groups 3 (*N* = 320) Healthy controls	Overall (*N* = 703)	*P* value
Age at scan, mean + SD		68.49 + 15.87	71.42 + 13.18	70.58 + 14.35	70.78 + 13.98	0.297^a^
Sex	Male, *N* (%)	27 (43)	144(45)	141 (44)	312(44)	>0.9^b^
Sex	Female, *N*(%)	36 (57)	176 (55)	179 (56)	391 (56)	
Race	non-white	12 (19)	39(12)	47(15)	98 (14)	0.3^b^
Race	white	51(81)	278(88)	273(85)	602 (86)	
Ethnicity	Non-Hispanic	54(86)	281 (88)	304 (95)	639 (91)	***0***.***002***^b^
	Hispanic	9 (14)	39 (12)	15 (5.0)	64 (9.1)	
Education		16 (12,18)	16 (12,18)	16 (14,18)	16 (13,18)	***<0***.***001***^c^

SD, Standard deviation.

^a^Anova.

^b^Pearsons Chi Square test.

^c^Wilcoxon Rank Sum test.

Statistically significant *P*-values are bolded and in italics.

**Table 2:** Dementia subtypes and cognition of groups 1 and 2

	Group 1 (*N* = 63) Seizures + Dementia/MCI	Group 2 (*N* = 320) Dementia/MCI only	*P* value
Type of dementia, AD	35 (56)	183 (57)	0.92^a^
Type of Dementia, non-AD	28 (44)	137 (43)	
CDR-global median	0.5 (0.5, 1)	0.5 (0.5, 1)	0.71^b^
CDR-SOB, median	2 (0.5, 5.3)	2.5 (1, 5)	0.81^b^

^a^Chi-squared.

^b^Wilcoxon rank-sum test between groups 1 and 2.

### Mesial temporal shape asymmetry in ADRD with and without epilepsy

AD_Epi versus AD_NoEpi and nonAD_Epi versus nonAD_NoEpi models were adjusted for covariates of age, sex, total intracranial volumes and dementia severity at the nearest visit measured via CDR-global.

We found that the nonAD_Epi, compared with nonAD_NoEpi (Fig. 2B), had left-to-right (left smaller than right) hippocampal head asymmetry (33 statistically significant points).

AD_Epi had no significant differences in hippocampal or amygdalar shape asymmetry compared with AD_NoEpi (Fig. 2A and C). No significant amygdalar asymmetry was observed between nonAD_Epi and nonAD_NoEpi (Fig. 2D).

In sensitivity analyses, AD_Epi versus AD_NoEpi and nonAD_Epi versus nonAD_NoEpi models were adjusted for MRI field strength and voxel volume in addition to age, sex, total intracranial volumes and dementia severity (Supplemental Fig. 1). NonAD_Epi, compared with nonAD_NoEpi, retained left-to-right (left smaller than right) hippocampal head asymmetry (39 statistically significant points). AD_Epi had no significant differences in hippocampal or amygdalar shape asymmetry compared with AD_NoEpi. No significant amygdalar asymmetry was observed between nonAD_Epi and nonAD_NoEpi.

### Mesial temporal shape asymmetry in ADRD with epilepsy compared with healthy controls

AD_Epi versus HC and nonAD_Epi versus HC comparison linear models were adjusted for covariates of age, sex and total intracranial volumes.

AD_Epi had left-to-right (left smaller than right) hippocampal tail asymmetry compared with HC (Fig. 3A).

The nonAD_Epi compared with HC (Fig. 3B) had an even more profound left-to-right (left < right) hippocampal head asymmetry (38 statistically significant points).

Neither AD_Epi nor nonAD_Epi had any significant differences in amygdalar shape asymmetry compared with HC (Fig. 3C and D).

In sensitivity analyses, AD_Epi versus HC and nonAD_Epi versus HC models were adjusted for MRI field strength and voxel volume in addition to age, sex and total intracranial volumes (Supplemental Fig. 2). AD_Epi had left-to-right (left smaller than right) hippocampal tail asymmetry compared with HC. The nonAD_Epi compared with HC had an even more profound left-to-right (left < right) hippocampal head asymmetry (31 statistically significant points). Neither AD_Epi nor nonAD_Epi had any significant differences in amygdalar shape asymmetry compared with HC.

### Mesial temporal shape asymmetry in ADRD without epilepsy compared with healthy controls

AD_NoEpi versus HC and nonAD_NoEpi versus HC comparison linear models were adjusted for covariates of age, sex and total intracranial volumes.

After adjusting for covariates of age, sex and total intracranial volumes, AD_NoEpi had left-to-right (Left < right) hippocampal head and tail asymmetry (10 statistically significant points in the head and one in the tail) compared with HC (Fig. 4A). Similarly, amygdalar shape asymmetry was observed in AD_NoEpi (22 statistically significant points, right < left) compared with HC (Fig. 4C).

After adjusting for covariates, nonAD_NoEpi had no significant differences in hippocampal or amygdalar shape asymmetry compared with HC (Fig. 4B and D).

In sensitivity analyses, AD_NoEpi versus HC and nonAD_NoEpi versus HC models were adjusted for MRI field strength and voxel volume in addition to age, sex and total intracranial volumes (Supplemental Fig. 3). AD_NoEpi had left-to-right (Left < right) hippocampal head asymmetry (3 statistically significant points) compared with HC. Similarly, amygdalar shape asymmetry was observed in AD_NoEpi (13 statistically significant points, right < left) compared with HC. After adjusting for covariates, nonAD_NoEpi had no significant differences in hippocampal or amygdalar shape asymmetry compared with HC.

### Healthy controls baseline hemispheric asymmetry

After adjusting for covariates of age, sex and total intracranial volume, HC showed left-to-right (Left < right) hippocampal head (Supplemental Fig. 4) asymmetry (10 statistically significant points) and amygdalar shape asymmetry (1 statistically significant point, right < left). More importantly, in sensitivity analysis, after adjusting the model for additional covariates of MRI field strength and voxel volume, healthy controls no longer demonstrated significant hippocampal asymmetry and only a single statistically significant point remained in the amygdala (Supplemental Fig. 5).

### Volume asymmetries

While overall left hippocampal volumes were slightly smaller than right among all groups, no statistically significant volume asymmetry differences were noted among any comparison groups after adjusting for covariates of age, sex, dementia severity and total intracranial volumes. (Table 3).

**Table 3:** Volume asymmetry among the groups

	Group A	Group B	Unadjusted *P*-value	Adjusted *P*-value
	AD + Epi	AD-Epi		
Hippocampal Volume Asymmetry, mean + SD	−0.004 + 0.080	−0.018 + 0.065	0.333	0.235
Amygdala Volume Asymmetry, mean + SD	−0.090 + 0.068	−0.085 + 0.109	0.684	0.928

	NonAD + Epi	NonAD-Epi		
Hippocampal Volume Asymmetry, mean + SD	−0.006 + 0.072	−0.008 + 0.075	0.877	0.557
Amygdala Volume Asymmetry, mean + SD	−0.047 + 0.146	−0.056 + 0.092	0.754	0.629

	AD + Epi	HC		
Hippocampal Volume Asymmetry, mean + SD	−0.004 + 0.080	−0.015 + 0065	0.423	0.350
Amygdala Volume Asymmetry, mean + SD	−0.090 + 0.068	−0.065 + 0.097	***0***.***049***	0.145

	NonAD + Epi	HC		
Hippocampal Volume Asymmetry, mean + SD	−0.006 + 0.072	−0.015 + 0065	0.514	0.405
Amygdala Volume Asymmetry, mean + SD	−0.047 + 0.146	−0.065 + 0.097	0.527	0.658

	AD-Epi	HC		
Hippocampal Volume Asymmetry, mean + SD	−0.018 + 0.065	−0.015 + 0065	0.649	0.692
Amygdala Volume Asymmetry, mean + SD	−0.085 + 0.109	−0.065 + 0.097	***0***.***043***	0.131

	NonAD-Epi	HC		
Hippocampal Volume Asymmetry, mean + SD	−0.008 + 0.075	−0.015 + 0065	0.343	0.261
Amygdala Volume Asymmetry, mean + SD	−0.056 + 0.092	−0.065 + 0.097	0.347	0.536

Note: SD, Standard deviation; AD + Epi, Alzheimer’s Disease with Epilepsy; AD-Epi, Alzheimer’s disease without Epilepsy; NonAD + Epi, Non-Alzheimer’s disease with Epilepsy; NonAD-Epi, Non-Alzheimer’s disease without epilepsy.

Unadjusted *P* values are calculated via *t*-test comparing groups A and B. Adjusted *P* values are calculated from linear models, adjusting the models for age, sex, dementia severity and total intracranial volumes for AD + Epi versus A-Epi and NonAD + Epi versus NonAD-Epi. Comparisons between dementia groups and healthy controls were adjusted for age, sex and total intracranial volumes.

Statistically significant *P*-values are bolded and in italics.

We included scanner-related sensitivity analyses (by including covariates of MRI field strength and voxel size) for shape asymmetry because those findings were significant and required assessment of robustness. We did not repeat these analyses for volume asymmetry, as the primary findings were null.

## Discussion

The most marked shape asymmetries in our study were in the non-AD group; those with epilepsy had asymmetrically smaller left hippocampal head compared with the right, compared with both non-AD without epilepsy and healthy controls. In the case of AD, asymmetry associated with epilepsy (compared with healthy controls) was observed, with a smaller left hippocampal tail compared with the right hippocampal tail. The findings suggest that comorbid epilepsy in PWCI is associated with morphological shape changes in dominant-hemisphere mesial structures. Importantly, these findings remained robust in sensitivity analyses, which additionally adjusted for MRI acquisition parameters, including field strength and voxel volume.

Previous studies investigating hippocampal atrophy have shown that while the AD subtype demonstrates the most significant hippocampal atrophy,^37^ it is also present in frontotemporal dementia,^38^ vascular dementia, Lewy body dementia and mixed dementia.^37-39^ While some hippocampal asymmetry may be observed in neurodegenerative disorders, hippocampal atrophy appears more or less generalized, especially in bilateral medial and lateral regions.^19^ In comparison, in people with mesial temporal lobe epilepsy (MTLE), asymmetry predominates with unilateral hippocampal volume reduction seen in the hippocampal head and lateral body, ipsilateral to the seizure focus and hippocampal tail contralateral to the seizure focus.^19,40^ These morphological patterns of hippocampal changes have been studied separately for these two diseases. However, to our knowledge, ours is the first study investigating the impact of the intersection of the two disease processes on the shape morphological pattern and volume asymmetries of mesial temporal structure.

The more pronounced left hippocampal head asymmetry observed in our study was in non-AD with co-morbid epilepsy compared with non-AD without epilepsy. This could be the result of epilepsy having a more pronounced impact on the dominant hippocampus compared with non-AD dementias, leading to more significant architectural alterations in the presence of co-morbid epilepsy. Moreover, in epilepsy, the hippocampal head is more prominently atrophied compared with the body and tail.^41^ Additionally, the left hippocampus may be more vulnerable due to its dominant role in language and higher cognitive functions, making it more susceptible to injury from recurrent seizures. This lateralization effect is supported by studies showing a greater incidence of left hippocampal damage in epilepsy. Recent studies indicate that seizures originating in the left hemisphere are associated with prominent asymmetric volume reduction, with more significant left hippocampal volume loss than seizures originating in the right hemisphere, which tend to cause bilateral hippocampal atrophy.^42^ Right-sided hemispheric seizures can result in substantial volume reduction of the left hippocampus, whereas the reverse is not typically observed.^43^ These findings suggest a greater vulnerability of the left hippocampus to injury. Additionally, it is also possible that epilepsy is more prevalent in certain non-AD dementias, such as the left temporal variant of frontotemporal dementia (FTD), compared with the right temporal variant, contributing to more pronounced asymmetric changes in the left hippocampus. Thus, the combined impact of epilepsy and neurodegeneration on the left hippocampus could explain the more significant asymmetric alterations observed in our study. This highlights the importance of considering lateralized effects and the specific vulnerabilities of different hippocampal regions in patients with co-morbid neurological conditions.

While the hippocampus is most prominently atrophied in MTLE, recent evidence has identified hippocampal damage and atrophy in both temporal and non-temporal newly diagnosed epilepsies. Therefore, hippocampal architectural alterations may be an indicator of epileptic activity regardless of epilepsy localization.^20^ Several mechanisms may account for the effects of active seizures on the hippocampus. These include neuronal loss, synaptic reorganization, neurogenesis and gliosis, alterations in neurotransmitter systems, oxidative stress, inflammation and network disruptions affecting hippocampal connectivity.^44,45^ Recurrent seizures often lead to significant neuronal loss, especially in the small granule cells of the dentate gyrus and CA1, CA2 and CA3 regions of the hippocampus, contributing to hippocampal sclerosis.^46,47^ The activation of NMDA receptors, Ca2+ influx, and an imbalance in Ca2+-binding proteins lead to neuronal hyperexcitability. This, along with inflammation and epigenetic regulation, leads to hippocampal damage.^48^ Recurrent seizures lead to neurogenesis, mossy fibre sprouting, gliosis and neurodegenerative processes, all of which damage the hippocampus.^46,49^ Studies have shown that patients with generalized epilepsy or epilepsy originating outside the temporal lobes can also exhibit hippocampal shrinkage.^50^ However, in extra-temporal epilepsy, the hippocampus atrophy is less pronounced than in MTLE.^20,51^ This might be due to widespread network disruptions affecting hippocampal connectivity and function rather than direct focal damage, as seen in MTLE.

We hypothesized that the co-existence of AD and epilepsy would result in a cumulative effect and, thus, more profound hippocampal morphological changes and volume asymmetries compared with AD alone. However, our findings did not support this hypothesis. A possible explanation is that the overlapping pathological effects of both diseases on the hippocampus might obscure distinct differences. Perhaps the additive insult of epilepsy is less pronounced in AD because the hippocampus is already severely involved, or the impact is temporal but extra-hippocampal. Additionally, the small sample size of AD participants with epilepsy may have limited our ability to detect statistically significant differences. It is also possible that other compensatory mechanisms in the brain could mitigate the additive effects of these diseases on hippocampal morphology.

Smaller amygdalar volumes have been demonstrated in both late-onset epilepsy of unknown aetiology and AD.^18^ However, our study is unprecedented in examining amygdala shape asymmetry in epilepsy and co-morbid neurodegenerative disorders. While we did not find any shape asymmetry in the amygdala in any of the epilepsy groups, the AD without epilepsy group demonstrated amygdala shape asymmetry in our cohort.

Previous studies of volumetric asymmetries have shown that the left hippocampus is slightly smaller than the right hippocampus in HCs.^52^ To our knowledge, ours is the first study to demonstrate shape asymmetry among HCs, showing the specific region within the left hemisphere (the left hippocampal head) that is asymmetrically smaller than the right. While HCs demonstrated the expected baseline hemispheric asymmetry, the asymmetry patterns identified in the ADRD and epilepsy groups in our study differed from these normative differences and therefore represent true disease-related effects. Importantly, our primary analyses compared ADRD with and without epilepsy, independent of HC. Comparisons with HC were included to contextualize these findings and demonstrate that the observed asymmetry patterns exceed those expected from normal hemispheric variation.

Finally, in this study, we also externally validated our previous findings and re-demonstrated asymmetrically smaller left hippocampal head, along with subtle left hippocampal tail asymmetry in AD without epilepsy compared with healthy controls.^27^ We extended our previous findings with additional findings of amygdalar asymmetry in AD without epilepsy.

Our study's most significant strengths are its novel and robust statistical technique, propensity score matching of the comparison groups and the quantitative morphological shape asymmetry analysis. Our previous work demonstrated that conventional MRI volumetric analysis reveals limited information about the mesial temporal structures and their involvement in the disease process.^27^ In the current study, we further demonstrated that volumetric asymmetry is insufficient to capture subtle differences between comparison groups. In contrast, statistical shape analysis captures more detailed localized neuroanatomic variations, which can have greater clinical and prognostic significance. Therefore, by examining brain asymmetry through shape analysis, we could potentially better characterize and visualize the morphological asymmetric changes in structures of interest in individuals with ADRD and comorbid epilepsy.^27^ We also note that our shape asymmetry emphasizes the left-to-right directional nature of hippocampal asymmetry.

Our study has several limitations. Because of the cross-sectional nature of our study, causality cannot be established, and we were unable to understand how seizures may impact hippocampal morphology longitudinally in ADRD. Cognitive assessments utilized in our models were extracted from the nearest clinic visit. The duration difference between the MRI scan date and the nearest clinic visits was not uniform across participants, and how this may have impacted our findings remains unclear. We were not able to account for epilepsy lateralization or the lateralization of epilepsy pathology, as this information was not available. The information regarding handedness and hemispheric dominance was not included. Nearly all right-hemispheric dominant individuals and the vast majority of left-handed individuals are left-hemispheric dominant. However, how the lack of this information may have impacted the results remains unclear. The significantly asymmetrically smaller left hippocampal head or tail when epilepsy was co-morbid may represent a sample bias of more left hemispheric pathologies underlying the included pathologies. Data on the type, severity, frequency, seizure onset, seizure duration, aetiology of seizures and EEG findings were not available. Acute symptomatic seizures are common in this age group. However, the data did not include this information. The sample size for epilepsy participants was relatively small. Another limitation of analysing asymmetry is that bilaterally symmetric pathological alterations would be classified as normal. Therefore, in our previous work, we analysed mean left and right hippocampal and amygdalar volumes and morphology separately as well as combined measures, to identify these differences.^53^ The current study was designed to complement prior work and extend it by focusing specifically on within-subject asymmetry. It is also important to note that FreeSurfer-derived volumes can overestimate hippocampal size, as automated segmentation can lead to over-segmentation. However, because the same segmentation method was applied consistently across all participants, any systematic bias would be expected to affect groups equally. Moreover, as hippocampal asymmetry was calculated within each participant, both hippocampi would be similarly over-segmented, minimizing the impact on asymmetry measures and, therefore, on the overall findings. We analysed AD and non-AD, but further dementia subtypes within non-AD were not separately assessed because of a relatively smaller sample size of participants with epilepsy. AD and non-AD neurodegenerative disorders were clinically diagnosed and not based on biomarkers. Future studies are needed to understand neuroimaging findings of subtypes of non-AD neurodegenerative disorders with co-morbid epilepsy. Lastly, the ADRC centres are not representative of the broader population, thereby limiting the generalizability of our findings.

In summary, our study found that a more profound asymmetrically smaller left hippocampal head asymmetric compared with the right hippocampal head may be observed in non-AD neurodegenerative disorders and co-morbid epilepsy. Our study identifies the left hippocampal head as the most susceptible to neurological injury in the setting of non-AD and co-morbid epilepsy. These findings suggest that hippocampal morphological asymmetry may serve as a neuroimaging correlate of epilepsy in non-AD. However, there is a need to confirm these findings in longitudinal, multicentre, randomized controlled studies. Nevertheless, our study serves as the first step toward more extensive and longitudinal studies to identify neuroimaging biomarkers for ADRD with co-morbid epilepsy.

## Supplementary Material

fcag179_Supplementary_Data

## Data Availability

Data used to prepare this article were obtained from the National Alzheimer’s Coordinating Centre database. For up-to-date information on the study, visit https://naccdata.org/requesting-data/nacc-data. All data used in this study, as well as a data dictionary, are available on the website. Additional related documents, including the study protocol and assay methods, are also available. Data access can be requested on the website.
