# Supplementary material for: Mesial temporal shape asymmetry as a neuro-imaging correlate of epilepsy in mild cognitive impairment and dementia
Source: Brain Commun. 2026 May 18;8(3):fcag179. doi: 10.1093/braincomms/fcag179 (PMC13225267; doi:10.1093/braincomms/fcag179)

**Supplemental Table 1: Demographics and Cognition**

|  | | AD+Epi (N=35) | AD-Epi (N=183) | NonAD+Epi (N=28) | NonAD-Epi  (N=137) | Healthy Controls (N=320) |
| --- | --- | --- | --- | --- | --- | --- |
| Age at scan, median (IQR) | | 74 (66, 82) | 75 (65, 84) | 66 (54, 80) | 68 (57, 78) | 73 (64, 80) |
| Sex | Male, N (%) | 15 (43%) | 81 (44%) | 12 (43%) | 63 (46%) | 141 (44%) |
| Sex | Female, N (%) | 20 (57%) | 102 (56%) | 16 (57%) | 74 (54%) | 179 (56%) |
| Race | non-white | 6 (17%) | 17 (9.4%) | 6 (21%) | 22 (16%) | 47 (15%) |
| Race | white | 29 (83%) | 164 (91%) | 22 (79%) | 114 (84%) | 273 (85%) |
| Ethnicity | Non-Hispanic | 31 (89%) | 164 (90%) | 23 (82%) | 117 (85%) | 304 (95%) |
|  | Hispanic | 4 (11%) | 19 (10%) | 5 (18%) | 20 (15%) | 16 (5.0%) |
| Education | | 16 (12, 18) | 16 (12, 18) | 16 (12, 18) | 14 (12, 17) | 16 (14, 18) |
| CDR global median (IQR) | | 0.5(0.5,1.5) | 0.5(0.5,1) | 0.5 (0,0.5) | 0.5 (0.5,0.5) | 0(0,0) |
| CDR-SOB, median (IQR) | | 4.5 (1.8, 7.5) | 3.5 (1.5, 6.0) | 1.0 (0.0, 3.0) | 1.0 (0.5, 3.0) | 0.0 (0.0, 0.0) |

IQR=Interquartile range, AD+Epi= Alzheimer’s Disease with Epilepsy, AD-Epi=Alzheimer’s disease without Epilepsy, NonAD+Epi= non-Alzheimer’s disease with Epilepsy, NonAD-Epi=non-Alzheimer’s disease without epilepsy

**Supplemental Table 2: Non-Alzheimer’s Disease Dementia subtypes**

|  | **NonAD-Epi** | **NonAD+Epi** | **p-value** |
| --- | --- | --- | --- |
| Vascular | 15 | 3 | 0.7 |
| Frontotemporal | 20 | 3 |  |
| Lewy Body | 15 | 1 |  |
| Mixed or others | 87 | 21 |  |

NonAD+Epi= Non-Alzheimer’s disease with Epilepsy, NonAD-Epi=Non-Alzheimer’s disease without epilepsy

**Supplemental Figure 1: Sensitivity Analyses of ADRD with and without epilepsy**

Multivariable linear models were used to compare shape asymmetry among groups after adjusting for age, sex, total intracranial volume, dementia severity, MRI field strength, and voxel Volume in sensitivity analyses. The green colour corresponds to positive values and implies that left-sided structures are larger than right-sided structures. Purple colour corresponds to negative values and implies that left-sided structures are smaller than right-sided structures. Statistically significant points are shown as dots on the figures. All visualisations are provided on the left-sided structures. **(1A)**: AD (Alzheimer’s Disease) with epilepsy is compared with AD without epilepsy, identifying no significant differences in the hippocampal shape asymmetry.

**(1B)**: Non-AD (Non-Alzheimer’s Disease) with epilepsy is compared with non-AD without epilepsy, identifying 39 significant points demonstrating hippocampal shape asymmetry with smaller left hippocampal head compared to right in the non-AD with epilepsy group.

**(1C)**: AD with epilepsy is compared to AD without epilepsy, identifying no significant shape asymmetry in the amygdala.

**(1D):** Non-AD with epilepsy is compared with non-AD without epilepsy, identifying no significant shape asymmetry in the amygdala.


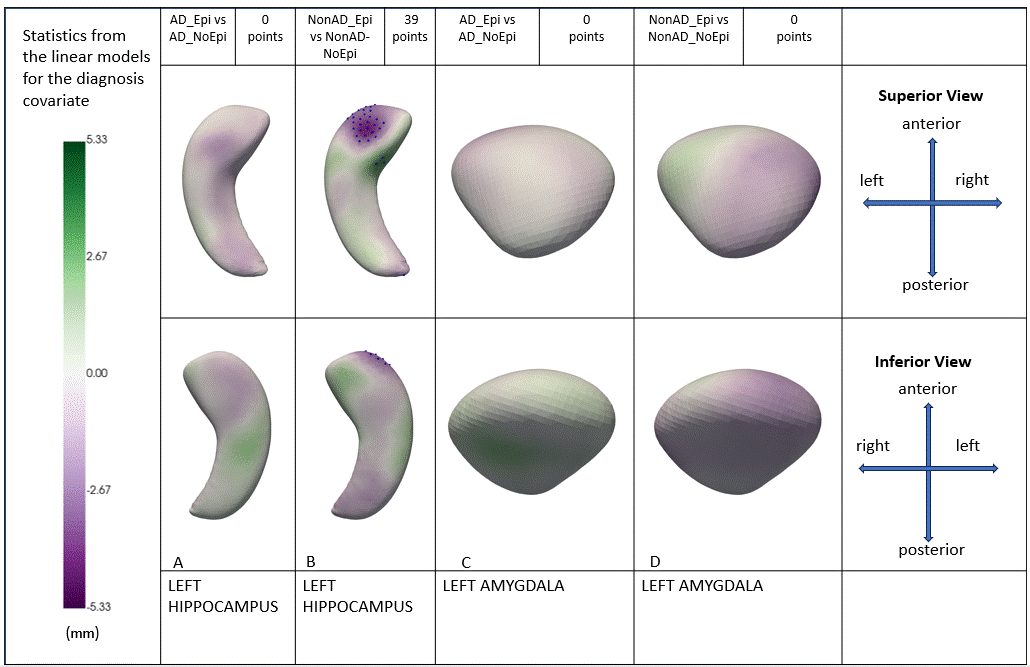


**Supplemental Figure 2: ADRD with Epilepsy compared to Healthy Controls**

Multivariable linear models were used to compare shape asymmetry among groups after adjusting for age, sex, total intracranial volume, dementia severity, MRI field strength, and voxel Volume in sensitivity analyses. The green colour corresponds to positive values and implies that left-sided structures are larger than right-sided structures. Purple colour corresponds to negative values and implies that left-sided structures are smaller than right-sided structures. Statistically significant points are shown as dots on the figures. All visualisations are provided on the left-sided structures.

**(2A)**: AD (Alzheimer’s Disease) with epilepsy is compared with healthy controls, identifying one significant point demonstrating a small left hippocampal tail compared to the right in the AD with epilepsy group.

**(2B)**: Non-AD (Non-Alzheimer’s Disease) with epilepsy is compared with healthy controls, identifying 31 significant points demonstrating hippocampal shape asymmetry with a smaller left hippocampal head compared to the right in the non-AD with epilepsy group.

**(2C)**: AD with epilepsy is compared to healthy controls, identifying no significant shape asymmetry in the amygdala.

**(2D):** Non-AD with epilepsy is compared with healthy controls, identifying no significant shape asymmetry in the amygdala.


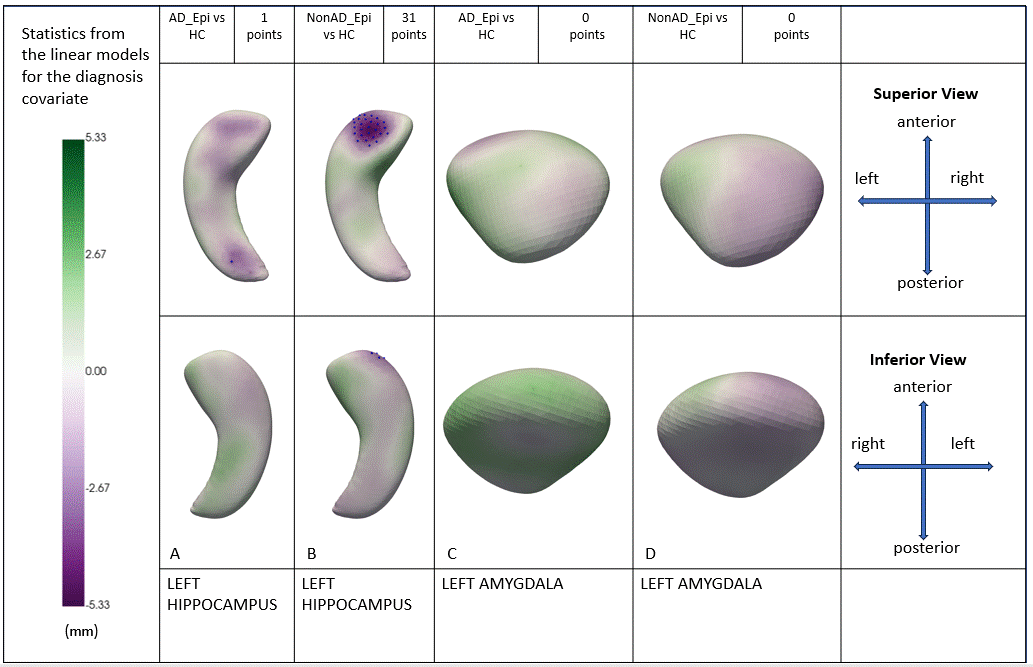


**Supplemental Figure 3: ADRD without Epilepsy compared to Healthy Controls**

Multivariable linear models were used to compare shape asymmetry among groups after adjusting for age, sex, total intracranial volume, dementia severity, MRI field strength, and voxel volume in sensitivity analyses. The green colour corresponds to positive values and implies that left-sided structures are larger than right-sided structures. Purple colour corresponds to negative values and implies that left-sided structures are smaller than right-sided structures. Statistically significant points are shown as dots on the figures. All visualisations are provided on the left-sided structures.

**(3A)**: AD (Alzheimer’s Disease) without epilepsy is compared with healthy controls, identifying 3 significant points demonstrating a small left hippocampal head compared to the right in the AD without epilepsy group.

**(3B)**: Non-AD (Non-Alzheimer’s Disease) without epilepsy is compared with healthy controls, identifying no significant shape asymmetry in the hippocampus.

**(3C)**: AD without epilepsy is compared to healthy controls, identifying 13 significant points demonstrating a smaller right amygdala compared to the left in AD without epilepsy.

**(3D):** Non-AD without epilepsy is compared with healthy controls, identifying no significant shape asymmetry in the amygdala.


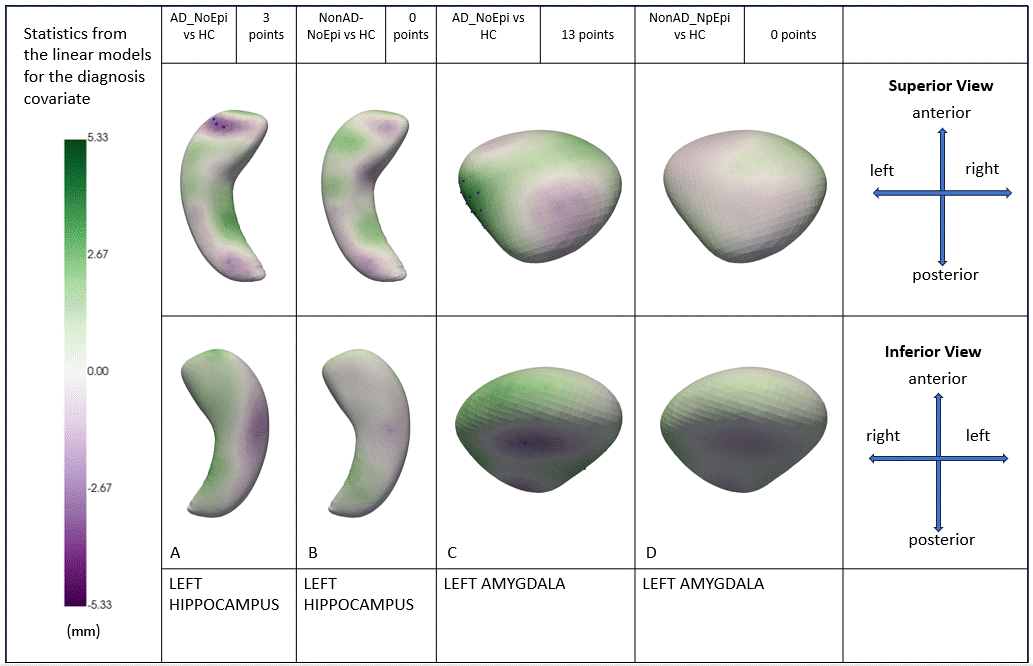


**Supplemental Figure 4: Healthy Control Asymmetry**

Multivariable linear models were used to investigate baseline shape asymmetry in the healthy control group. Analyses are adjusted for covariates of age, sex, and total intracranial volume.

The green colour corresponds to positive values and implies that left-sided structures are larger than right-sided structures. Purple colour corresponds to negative values and implies that left-sided structures are smaller than right-sided structures. Statistically significant points are shown as dots on the figures. All visualisations are provided on the left-sided structures.

**4A)** Healthy controls showed left-to-right (Left < right) hippocampal head asymmetry (10 statistically significant points)

**4B)** Healthy controls showed left-to-right amygdalar shape asymmetry with only a single statistically significant point (1 statistically significant point, right <left).


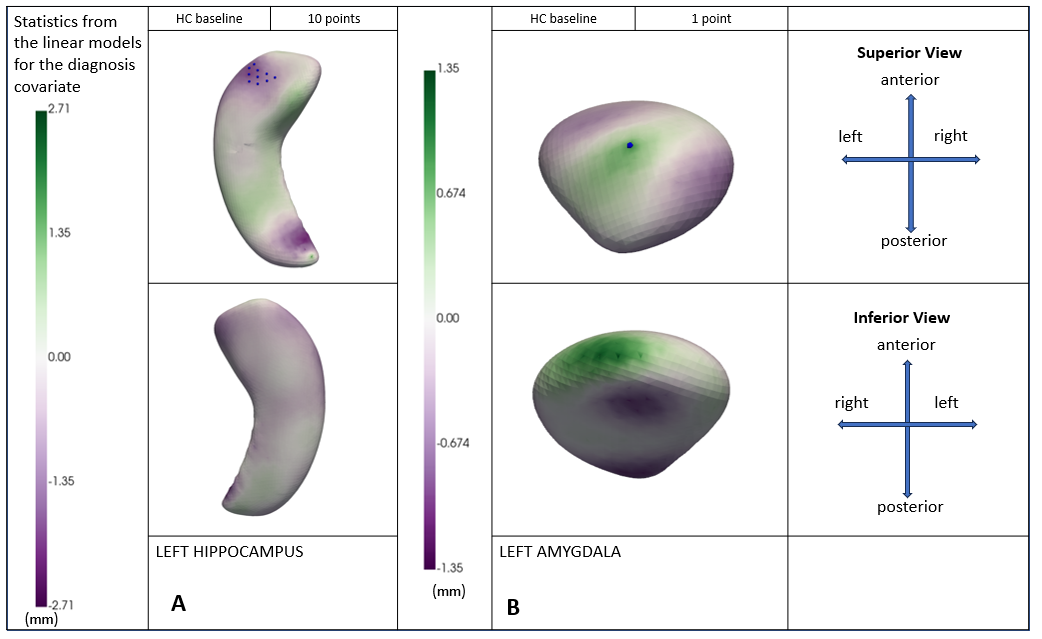


**Supplemental Figure 5: Sensitivity Analysis of Healthy Control Asymmetry**

Multivariable linear models were used to investigate baseline shape asymmetry in the healthy control group. Analyses are adjusted for covariates of age, sex, total intracranial volume, MRI field strength, and voxel volume.

The green colour corresponds to positive values and implies that left-sided structures are larger than right-sided structures. Purple colour corresponds to negative values and implies that left-sided structures are smaller than right-sided structures. Statistically significant points are shown as dots on the figures. All visualisations are provided on the left-sided structures.

**5A)** Healthy controls no longer demonstrated hippocampal asymmetry

**5 B)** Healthy controls showed only a single statistically significant point in the amygdala (right <left).


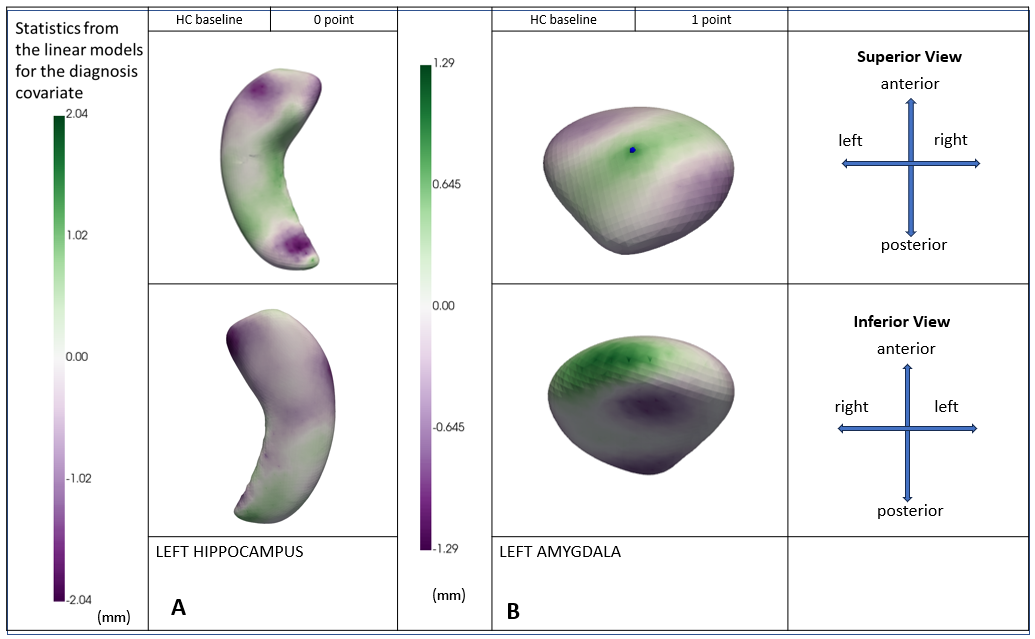

Supplement: fcag179_Supplementary_Data [file fcag179_supplementary_data.zip › Supplementary_Tables_and Figures.docx]
